# The Aim and Working of a Festivities Fund

**Published:** 1913-01-04

**Authors:** James Henry Smethurst

**Affiliations:** Chairman of the Board.


### THE AIM AND WORKING OF A FESTIVITIES FUND.

### A Lesson from the Warrington Infirmary.

By JAMES HENRY SMETHURST, Chairman of the Board.

WHEN I read in THE HOSPITAL of December 28, 1912, your invitation to send an account of Christmas festivities in the various institutions similar to this, I thought that what was done here and the means taken to carry the work through might not only be of interest to your readers but also be a guide for others to follow. Before doing so may I transgress just a moment to say how much I appreciate THE HOSPITAL as a paper, and how grateful one ought to be for the clear way you are dealing at the present moment with that nightmare—the Insurance Act—and its effect upon the funds of such places as ours? For many years I have been interested in charitable work, but it is only seven years since I was placed on the management of this institution, which is now in its 104th year, and I shall never forget the first visit I paid on a Christmas Day to our wards. A certain amount of decoration had been done, and the committee had allowed the special Christmas fare, but somehow I felt that things were not right, and I made up my mind to do something more. The following year I wrote to some of my friends who did not subscribe to the general hospital funds, first of all, and then asked the board for a small subscription on the last meeting before Christmas. As a result I got £25, and provided a small Christmas tree, on which were placed useful presents, such as shirts for the men, shawls, etc., for the women, toys and chocolates for the children. Then I invited the subscribers to come and see the result. My reason for this was to stir up greater interest amongst people who had not been reminded in the past. Everything passed off well, and as our bed accommodation was raised from forty to eighty the following year I knew perfectly well that more money was wanted. Going into town on my week, as I have come to call it, I noticed that the monkey "Consul" was appearing at the Hippodrome. I wrote asking his manager to allow him to collect funds. The reply was: "Come yourself, and he will help you." I collected £8, and owing to the interest taken the result of the year was a sum of £45. With this I told the board that I would take over the whole of the expense of Christmas festivities, even down to paying the extra for electric light.

A larger Christmas tree was got, electrically lighted by our own electrician; better presents for the in-patients, and also something for every member of the nursing and general staff of the hospital. The following year found us with more patients than ever—plenty of ordinary financial worry—but with a very light heart I asked the management of the Hippodrome to allow me to go on the stage at each of their shows in the week to tell the people what I wanted. I was allowed three minutes to speak, then the ambulance men (who had volunteered to help me) collected, and as a result over £20 came from here alone.

The following year I wrote to every place of amusement to ask for help, and three agreed to my suggestion, and with the subscriptions obtained the total amount realised was £59. With this I not only did as in the previous year for the in-patients and staff, but provided a piano or gramophone for each ward, and gave afternoon tea to the visitors, who in their turn put something in the collecting boxes at the door of the hospital. By the way, I may say that all these useful boxes were banished from the wards during the week. In addition the nursing and general staff had a dance, to which they could invite a friend. Last year (1911) the work went on the same lines, except that we had another day for the children in our out-patient department and home patients. They came, had tea and small presents and chocolates, etc.

The picturedromes agreed to allow me to speak, and when I could not attend displayed a notice on the screen and the attendants collected. As a result £79 was realised, and whilst the cost of the festivities was £42, the balance was handed over to special hospital purposes, such as the Samaritan Fund, the "Children's Cot," and so forth.

This year I obtained the consent of every place of amusement—my old friends at the Hippodrome, two theatres, five picture palaces, and skating rink. I was very anxious to obtain a surplus of £65 to defray the balance of the cost of a recently installed *x*-ray appliance. I only finished my "star turns" at 11 o'clock last night, but I have the satisfaction of knowing that my object has been more than achieved, as £105 is in the bank, and some small amounts have still to come from the small places. Naturally a person strange to the work may ask, "Is it worth it?" I reply, "Certainly," many times over. Apart from the pleasure afforded, and life in hospital is a sad one at the best of times, it stirs up the interest of people, makes them better acquainted with the needs of the institution, and increases their kindly concern in all pertaining to it.

I am more than grateful to the general subscribers and givers of toys for all the help they give, and can never thank the public generally in this town for the way in which they have responded. I must have spoken to nearly 50,000 people, and not one word has been said against the method adopted.

I was amply repaid at the Hippodrome last night (December 28) when I went on the stage for the third time that evening and told the 2,000 people there that the total in that one place alone was £38 5s. 10d., and the audience cheered again and again.

I sincerely hope that no reader of this hurriedly written paper will think that what I have tried to do has been for "self-advertisement." I believe that all ought to help some cause for the betterment of their fellow-men, and this is the way I have chosen, with the measure of success described above.

